# University of Iowa

**Published:** 1888-07

**Authors:** 


### University of Iowa—Dental Department.

The sixth annual commencement of the Dental Department of the State University of Iowa was held at the Opera House, Iowa City, Iowa, on Monday, March 5, 1888, at 8 o'clock P. M.

The degree of D.D.S. was conferred on the following graduates by the President, Charles A. Schaeffer, Ph.D.

| NAME. | RESIDENCE. | NAME. | RESIDENCE. |
|---|---|---|---|
| A. E. Anger | Iowa. | H. V. McGregor | Iowa. |
| J. E. Babcock | Illinois. | M. S. Overfield | Iowa. |
| C. P. Beyer | Iowa. | C. S. Percival | Iowa. |
| F. T. Breene | Iowa. | A. B. Palmer | Iowa. |
| C. W. Cope | Iowa. | A. L. Punton | Iowa. |
| E. S. Dawson | Iowa. | L. E. Roe | Nebraska. |
| H. D. Hinkley | Iowa. | A. L. Rist | Iowa. |
| O. A. King | Iowa. | J. E. Swain | Iowa. |
| J. A. Lovelady | Iowa. | C. H. Sippel | Iowa. |
| J. A. Leonard | Iowa. | C. S. Searles | Iowa. |

